# The Zinc Finger Ran-Binding Protein 3 (ZRANB3): An Advanced Perspective

**DOI:** 10.3390/ijms27020574

**Published:** 2026-01-06

**Authors:** Paride Pelucchi, Ettore Mosca, Nika Tomsič, Yossma Waheed, Wendalina Tigani, Alice Chiodi, Aditya Mojumdar, Marco Gerdol, Matteo De March

**Affiliations:** 1 Istituto di Tecnologie Biomediche (ITB), Consiglio Nazionale delle Ricerche (CNR), Via Fratelli Cervi 93, 20054 Milano, Italy; paride.pelucchi@itb.cnr.it (P.P.); ettore.mosca@itb.cnr.it (E.M.);; 2 Laboratory for Environmental and Life Sciences, University of Nova Gorica, Vipavska 13, SI-5000 Nova Gorica, Slovenia; 3 Department of Life Science, University of Trieste, Via Licio Giorgieri 5, 34100 Trieste, Italy; 4 Department of Biochemistry and Microbiology, University of Victoria, Victoria, BC V8W 2Y2, Canada

**Keywords:** ATP-ase translocase, fork remodeler, DNA repair, tumour suppressor, cancer

## Abstract

Human zinc finger Ran-binding protein 3 (ZRANB3) is crucial for DNA damage tolerance (DDT), as it prevents excessive damage, restores fork progression, and ultimately maintains genome stability. This unique and ancient architecture mainly exerts its function during replication fork reversal (RFR) and within the p53/Polι axis; thus, ZRANB3 is considered a tumour suppressor. However, possible additional roles in DNA synthesis and cell metabolism have been proposed. In tumour cells, *ZRANB3* gene expression is deregulated, a condition that is frequently associated with poor survival and adverse clinical outcomes. *ZRANB3* can be altered by functional mutations, gene copy number alterations, and a combination of the two. Although its mRNA levels typically correlate with p53 expression, this correlation breaks down in the context of p53 mutations and high proliferative activity. This comprehensive review integrates the currently available yet fragmented literature on ZRANB3, both at the gene and protein levels, examines its regulation in cancer development, and discusses the evidence supporting its role as a tumour suppressor and prognostic biomarker.

## 1. Introduction

Cancer is a multifactorial disease characterised by uncontrolled cell growth and proliferation, which is influenced by genetic and environmental factors. Among genetic components, tumour suppressor genes play a crucial role in preventing the development of this disease, as they often encode proteins that regulate cell growth, repair DNA damage, and/or induce apoptosis when abnormal cells are detected. One of these genes is *TP53* [1,2,3]. Either specific mutations on *TP53* or loss of protein function are frequently found in human cancers, indicating that mutant p53 forms promote tumour development, thus providing strong genetic evidence of its physiological function [4,5]. Other functionally similar known proteins are breast cancer types 1 and 2 (BRCA1 and BRCA2), which both contribute to cell cycle and genome stability by participating primarily in homologous recombination (HR) repair of DNA double-strand breaks (DSBs) [6]. These genes are best known because their dysfunction is strongly linked to breast and ovarian cancers [7,8].

Indeed, cancer can be triggered by DNA damages that, if not resolved, can lead to more serious consequences, such as DSBs, by hindering the progression of replication machineries and generating inherently unstable stalled forks, which are consequently prone to structural collapse and aberrant rearrangements [9,10]. Therefore, in order to restore normal DNA replication and cell turnover, these DNA hotspots need to be accurately processed and restarted.

DNA damage tolerance (DDT) pathways pause DNA synthesis to facilitate the bypassing of DNA lesions and aid in fine-tuning the restarting of fork speed [11]. In this context, replication fork reversal (RFR) converts the original three-way forks into a 4-way junction by reannealing the newly synthesised strand with the parental templates, a complex and not yet fully understood mechanism that requires the coordinated action of different players [12,13,14,15].

The same BRCA1/2 proteins stabilise the regressed arms and prevent nascent DNA degradation by nucleases [16,17,18]. This action is exerted in association with DNA repair protein RAD51, which, in turn, competes for binding to DNA with ssDNA-binding replication proteins A (RPAs)—responsible for preventing local DNA reannealing and fork collapse—and promotes fork reversal through its strand exchange activity [19,20,21,22]. However, because the activity of RAD51 may induce excessive fork reversal, it should be finely modulated by RECQ-like helicase 5 (RECQL5), which acts to limit this process, allowing DNA synthesis to continue and preventing premature fork stalling [23]. Notably, RECQL5 belongs to the same RECQ family as RECQ1, which was the first helicase to be characterised in fork reversal [24]. However, in RECQL5-deficient cells only, the expression of HR-defective RAD51 mutants was shown to rescue replication defects in the absence of DNA translocases, supporting an additional functional interconnection during this process [23].

In fact, RAD51 is also linked to three SNF2-family chromatin remodelers: SWI/SNF-related matrix-associated actin-dependent regulator of chromatin subfamily A-like protein 1 (SMARCAL1); its cousin, zinc finger Ran-binding protein 3 (ZRANB3); and helicase-like transcription factor (HLTF). Their activity in *BRCA1/2*-deficient cells leads to excessive nascent strand degradation and consequently genome instability [25,26,27,28].

SMARCAL1, ZRANB3, and HLTF are all essential for protecting stalled forks and maintaining genome stability, which is why they are considered oncosuppressors [29,30,31,32].

However, while SMARCAL1 and HLTF may have dual roles in cancer, ZRANB3 function appears to be more conservative in this regard, especially after the discovery of a ZRANB3/p53 axis during DNA damage repair [28,33,34,35].

## 2. Evolution of *ZRANB3* Gene

The *ZRANB3* gene is strongly evolutionary conserved across metazoans, as a single copy of *ZRANB3* orthologs is present in >95% of sequenced animal genomes, with occasional duplication events restricted to allotetraploid organisms or organisms that underwent whole genome duplication [36,37,38] (Supplementary Material—phylogenetic analysis [39,40,41,42,43,44,45,46]). Remarkably, *ZRANB3* orthologs are present in most basal animal phyla (including Porifera, Ctenophora, and Cnidaria, but excluding Placozoa), and the evolutionary origin of this gene can be traced by inference to a time point prior to the most recent ancestor of Choanozoa, i.e., 950 Mya, as revealed by the presence of unequivocal 1:1 orthologs in the genomes of choanoflagellates *Monosiga brevicollis* and *Salpingoeca rosetta* (Supplementary Figure S1A—evolutionary tree of *ZRANB3*).

Interestingly, the split between *ZRANB3* and the closest vertebrate paralog, *SMARCAL1*, occurred in the same timeframe [47]. However, while *SMARCAL1*, which lacks orthologs in other eukaryotic taxa, most likely represents a choanozoan innovation, *ZRANB3* appears to have a much more ancient origin. In fact, the presence of *ZRANB3*-like genes in several non-metazoan eukaryotic phyla (i.e., Stramenopiles, Alveolata, Haptista, Apusozoa, and Viridiplantae) suggests that a prototypical *ZRANB3*-like gene was already present in the latest common ancestor of all eukaryotes (Supplementary Material, Supplementary Figure S1A). Moreover, although these genes encode protein sequences with rather poor homology to their counterparts in metazoans (~30%), they invariably contain both the N-terminal motor ATP-ase core and the nuclease domain (Supplementary Material). The latter is, indeed, widespread in nature and it is often associated with multidomain proteins [48].

Therefore, a variety of different genes encoding this functional domain may have served as donors for the origin of *ZRANB3* and *ZRANB3*-like genes [49,50].

## 3. ZRANB3 Protein Structure and Function

### 3.1. Structural and Biochemical Properties

**Figure 1:**
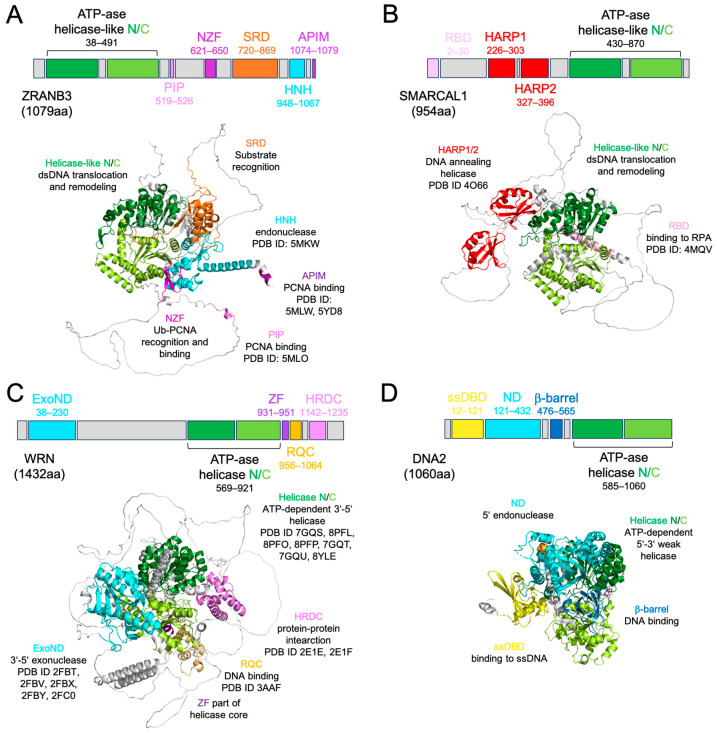
Domain and structural organisation of ZRANB3 protein compared to SMARCAL1, WRN, and DNA2. (**A**) Human ZRANB3 full-length (FL) protein (ATP-ase helicase-like N/C: N-terminal and C-terminal ATP-dependent helicase-like motor domains; PIP: PCNA-interacting domain; NZF: zinc finger RANBP-2 type domain; SRD: substrate recognition domain; HNH: nuclease domain; APIM: AlkB homologue 2 PCNA-interacting motif) and its Alphafold model (same colour code); the PDB ID codes and functions of each domain are also reported. (**B**) Human SMARCAL1 FL protein (RBD: RPA-binding domain; HARP1/2: hepa-A-related protein domain; ATP-ase helicase-like N/C: N-terminal and C-terminal ATP-dependent helicase-like motor domains) and its Alphafold model (same colour code); the PDB ID codes and functions of each domain are also reported. For consistency, we used the same structural orientation as in panel (**A**) by overlapping the ATP-ase core of SMARCAL1 to the same conserved domain of ZRANB3. (**C**) Human WRN FL protein (ExoND: exonuclease domain; ATP-ase helicase N/C: N-terminal and C-terminal ATP-dependent helicase domain; ZF: zinc finger; RQC: RecQ C-terminal domain; HRDC: helicase and RNaseD C-terminal domain) and its Alphafold model (same colour code) in the same orientation as in panel (**A**); the PDB ID codes and functions of each domain are also reported. (**D**) Human DNA2 FL protein (ssDBD: ssDNA binding domain; ND: nuclease domain; b-barrel domain; ATP-ase helicase N/C: N-terminal and C-terminal ATP-dependent helicase domain) and its Alphafold model (same colour code) in the same orientation as in panel (**A**); the PDB ID codes and functions of each domain are also reported.

The human *ZRANB3* gene encodes a 1079-residue protein, which is characterised by the presence of 6 functional domains [27]: the ATP-ase N- and C-terminal motor-remodeling domain, the PCNA-interacting protein domain (PIP), the NPL4 zinc finger motif (NZF), the substrate recognition domain (SRD), the His-Asn-His (HNH) nuclease domain, and the AlkB homologue 2 PCNA-interacting motif (APIM) (Figure 1A).

Compared to SMARCAL1 and to the helicases-nucleases that share similar features and common pathways with ZRANB3, namely WRN RecQ-like helicase (WRN) and DNA replication helicase/nuclease 2 nuclease (DNA2), ZRANB3 shows the ATP-ase motor domain at the N-terminus (Figure 1A–D) [51,52,53]. This entire module is indeed structurally conserved among DExH-box helicases, such as WRN, but in ZRANB3 as well as in SMARCAL1 it catalyses ATP-dependent DNA translocation, while in WRN and DNA2, which is SF1-helicase, it unwinds DNA [27,54,55,56,57] (Supplementary Table S1—structural and functional details of ZRANB3, SMARCAL1, WRN, and DNA2). Uniquely, SMARCAL1 can anneal DNA through HARP [58]. The motor domain of ZRANB3 has two helical portions, HD1 and HD2, conserved in eukaryotic Rad54 and in human HLTF, which confer additional remodeling capability (ATP-ase_RDs) [59,60,61]. Mutations in ATP-ase_RD were shown to compromise ZRANB3 function on fork DNA structures [62]. Noteworthily, ZRANB3, as well as SMARCAL1, WRN, and DNA2, can remodel R-loops in vitro, even though the molecular details of this complex process remain unclear in absence of structural data (Supplementary Table S1) [63,64,65].

According to the general mechanistic view of motor remodeling action, the most critical homology-based ZRANB3 DNA-binding residues (Q114, Y132, R169, E197, and R237) lie on the N-lobe of this core, which directly binds DNA and coordinates protein translocation with the help of the more auxiliary C-lobe (Supplementary Material and Supplementary Figure S1 [66,67,68,69,70,71,72,73]) [61,74]. Notably, Arg226, which is mutated in colorectal cancer (CRC), belongs to a β-hairpin adjacent to the HD1 insertion and its configuration may support the involvement of ZRANB3 in nucleotide excision repair (NER) [75,76].

Altogether, these data highlight the importance of the ATP-dependent motor domain for ZRANB3 function, which interestingly can be fully exerted only in presence of conserved SRD (N720 to T869) [32,62]. At the same time, SRD facilitates binding to specific DNA structures, particularly splayed arms, but has no affinity alone for either ssDNA or dsDNA substrates and only reduced affinity for splayed-arm DNA compared to the full-length protein [32,62] (Figure 1A, Supplementary Table S1). Additionally, SRD mutants (L760A, D761A, and I762A) displayed a clear deficiency in DNA-stimulated ATP-ase activity, confirming the functional inter-dependence of these two domains for ZRANB3.

Notably, SMARCAL1, which lacks SRD, exhibits similar preferences to ZRANB3 for splayed arms (Figure 1B, Supplementary Table S1), providing evidence of their structural divergence but partial functional overlap [77]. Indeed, ZRANB3 cleaves 5′-flap DNA ends with its HNH (S948 to K1067), while SMARCAL1 lacks nuclease activity [27,54]. By comparison, WRN and DNA2 show 3′-5′ exonuclease and mostly endonuclease activity on 5′-ends, respectively (Figure 1C,D and Supplementary Table S1) [51,78]. ZRANB3 HNH mutants ∆975–1013 and ∆972–1010 were unable to catalyse the endonucleolytic reaction and could partially impair ATP-ase activity, suggesting that even these two modules may work cooperatively [27]. By contrast, double mutant K1046A/R1048A and α8-R1009A exhibited significantly reduced endonuclease activity while maintaining ATP-ase function [27], indicating that the HNH alone is sufficient for DNA cleavage.

Therefore, the ATP-ase core, SRD, and HNH are functionally interconnected and essential for ZRANB3 activity, and mutations affecting any of these domains can compromise overall protein function. Table 1 reports in full the biochemical properties of ZRANB3 exerted both on DNA substrates and on the proliferating cell nuclear antigen sliding clamp (PCNA).

In fact, ZRANB3 interacts with PCNA through the PIP-box (Q519-F526), as mutations in its consensus sequence Q-x-x-[VILM]-x-x-[FY]-[FY] were shown to abolish ZRANB3–PCNA complex formation [27,54,79] (Figure 1A, Table 1). Curiously, this interaction can also occur through APIM, the second well-conserved PCNA-interacting region at the extreme ZRANB3 C-terminus [80]. Additionally, Zn finger NZF (P621 to S650), located in between, serves as a third PCNA anchoring point, specifically for its K63-ubiquitinated version [54,79] (Figure 1A, Table 1). Mutations in this domain completely abolished the recruitment of ZRANB3 to stalled forks (Table 1). By comparison, SMARCAL1 is recruited to the site of lesion through the RBD domain, while WRN is recruited through its HRDC (Figure 1B,C) [81,82]. The presence of these three distinct PCNA-interacting modules in ZRANB3 highlights the importance of this protein in PCNA-mediated pathways.

### 3.2. ZRANB3 Molecular Mechanisms: RFR and Beyond?

ZRANB3 has a pronounced preference for binding to poly-ubiquitinated PCNA compared to its mono-ubiquitinated forms, which confirms its primary role in damage repair and particularly RFR [54]. In this sense, it was demonstrated that *ZRANB3*-KO cells were shown to exhibit unrestrained fork progression in response to several chemical treatments. Specifically, PIP+APIM mutants were shown to be defective in fork slowing and reversal and failed to restore chromosome integrity after Camptothecin treatment (CPT), which caused DSB accumulation [30].

Moreover, depletion of the three translocases, including *ZRANB3*, as well as *RAD51* knockouts in *RTEL1*-depleted cells, which already experienced high levels of replication stress, led to increased abundance of DSBs compared to cells depleted of *RTEL1* only [83]. Similarly, the co-depletion of *ZRANB3* and *HLTF* in damaged *RECQL5*-depleted cells induced the rescue of replication defects [23]. Altogether, these observations support a central role for ZRANB3 in genome maintenance, which mainly occurs through PCNA-mediated interactions but may extend to other partners of the same regulatory network (Table 2) [84].

Interestingly, the same interaction with PCNA could confer additional function to ZRANB3. Specifically, the ZRANB3 C-terminal region (929–1079), which also includes APIM, was reported to bind the α-subunit of ribonucleotide reductase (RNR-α), which in turn antagonises ZRANB3 and inhibits the ZRANB3-driven DNA synthesis process [85]. In particular, nuclear RNR-α levels were shown to modulate the extent of ZRANB3′s association to PCNA, but not Ub-PCNA, in non-stressed cells as a defence mechanism against high levels of dNTPs [85]. This observation highlights the complex interconnections between these two proteins and supports a diverse yet poorly understood role for ZRANB3 in DNA synthesis.

Of particular note are the few ZRANB3 co-expressed partners responsible for maintaining and regulating centrosome dynamics, the centrosomal proteins (CEPs) [86] (Table 2). As co-expressed proteins often participate together in the same functional pathways, a possible involvement of ZRANB3 in cell division merits investigation. Curiously, such a role was reported for one of the close ZRANB3 relatives, the SNF2-family chromatin remodeler, BAF, as the absence of double PHD-finger subunit 3 (DPF3), which binds to histones, led to chromosome alignment defects and altered mitotic progression [87].

Despite these few exceptions, the ZRANB3/p53 axis is mostly recognised as playing a significant role in damage repair. High nuclear levels of p53 promote the formation of a complex with polymerase iota (POLι), triggering the slowdown of DNA replication through the activation of polyUb-PCNA-ZRANB3-dependent DDT fork remodeling pathways [34]. *ZRANB3*-knockdown cancer cells engineered to control p53WT expression showed increased track lengths at the maximal p53 level. A similar conclusion was obtained in undifferentiated cancer cell lines using double knockout for *Polι*/*p53* and *ZRANB3* [35].

During RFR, ZRANB3 is also known to interact with the ring finger and WD repeat domain 3 (RFWD3), which participates in cell cycle regulation together with p53 [88,89]. In fact, the depletion of *RFWD3* in those cells expressing p53 WT was shown to reduce the levels of the origin recognition machinery, even though this effect did not involve ZRANB3. Instead, co-depletion of ZRANB3 and RFWD3 in BRCA2-deficient cells, which are known to promote nascent DNA degradation due to excessive fork remodeling activity mediated by ZRANB3, suppressed fork collapse [19,88]. These observations support a role for the ZRANB3/RFWD3 axis in fork remodeling but not in p53-driven cell cycle regulation.

## 4. ZRANB3 in Cancer Development

Overall, ZRANB3 appears to be primarily dedicated to maintaining cellular integrity, although evidence supporting this role at cellular level remains limited.

One of these comes from *Myc*-driven B-cell lymphomas, where inactivation of *ZRANB3* impaired the capability of these cells to restore normal DNA replication by fork reversal and to suppress cancer development [90]. Curiously, *ZRANB3*-haploinsufficiency, but not *SMARCAL1*-haploinsufficiency, in the same cells had equal effects, suggesting a more critical involvement of ZRANB3 in resolving B-cell oncogene-induced stress than SMARCAL1 [90]. Other evidence links endometrial carcinoma (EC) to ZRANB3 loss-of-function, as several EC mutants in the ZRANB3 ATP-ase core and HNH domain showed both compromised ATP-ase and nuclease activities [27]. Similarly, *ZRANB3*-knockout hematopoietic stem and progenitor cells (HSPCs) resulted in cells with accumulated DNA damage and replication stresses [91].

Instead, it is noteworthy that NIH3T3 H-Ras^G12V^-transformed fibroblasts, which promote uncontrolled leukaemia cell proliferation, were shown to be reliant on ZRANB3-supported DNA synthesis for their growth [92]. This observation suggested on the opposite side a possible oncogene-associated role for ZRANB3 in these cells and its potential as a biomarker in leukaemia treatment [92]. As for SMARCAL1, a possible ZRANB3 cancer-associated duality is intriguing, albeit at present it appears confined to this tissue. Also, a possible alternative function in glucose metabolism merits attention, especially because it could reconnect ZRANB3 to pancreatic cancers, where also WRN and DNA2 were suggested as potential biomarkers [93,94,95].

To provide a more complete view of ZRANB3 regulation and its role in cancer, below we integrate and discuss the complete *ZRANB3* TCGA alterome and its correlation with p53 (Supplementary Information [68,96,97,98,99]), a section that remains speculative pending experimental validation.

### 4.1. The ZRANB3 TCGA Pan Cancer Alterome

**Figure 2:**
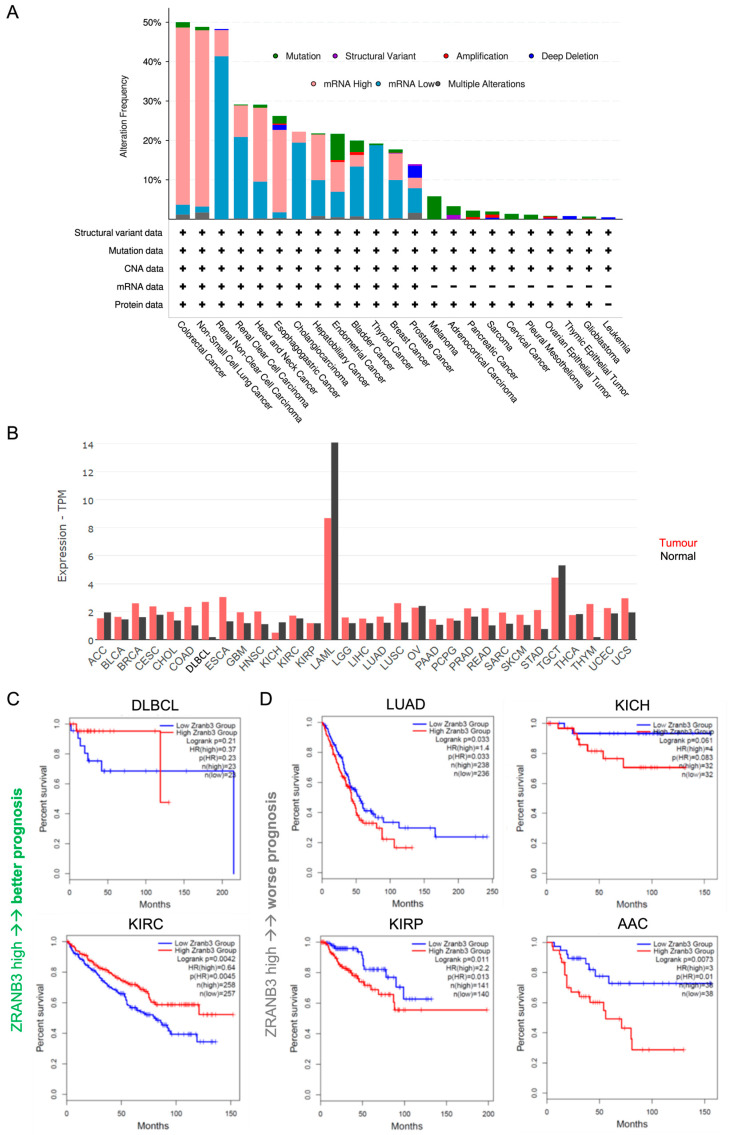
(**A**) Overall *ZRANB3* genetic alterations (%) in all tumours (mRNA data are compared to those found in normal cells). (**B**) *ZRANB3* mRNA expression in all tumours (red bars) compared to normal cells (black bars); each bar represents the median of *ZRANB3* mRNA expression level expressed in transcript per million (TPM). (**C**) Kaplan–Meier representation of overall survival with respect to *ZRANB3* mRNA expression. For each tumour type, the patient cohorts are divided into two equal sub-groups at the median of *ZRANB3* expression. Each plot shows the number of patients in each sub-group, the hazard ratio (HR) representing the difference of risk between the two groups (>1 if the risk is higher in the high-expressing group and <1 if the risk is lower), and the *p*-value resulting from a log-rank test (logrank p). DLBCL: diffuse large B-cell lymphoma; KIRC: kidney renal clear cell carcinoma. (**D**) Same plot as in panel (**C**) for lung adenocarcinoma (LUAD), kidney chromophobe (KICH), kidney renal papillary cell carcinoma (KIRP), and adrenocortical carcinoma (ACC).

In the majority of tumours, *ZRANB3* appears to be (i) abnormally expressed, either at high or low levels; (ii) mutated; or (iii) altered in copy number (copy number alterations, CNAs) (Figure 2A).

Compared to normal tissues, *ZRANB3* is more often upregulated than downregulated in cancer overall (Figure 2B). This is particularly observed in diffuse large B-cell lymphoma (DLBCL) and thymoma (THYM), but also in colon adenocarcinoma (COAD), esophageal carcinoma (ESCA), and lung squamous cell carcinoma (LUSC). Curiously, in DLBCL, *SMARCAL1* was found to be mutated, a condition that may require counteraction of ZRANB3 to balance its dysfunctional effect [100]. The same hypothesis might also be extended in this tumour to ZRANB3 in relation with WRN, as mutations in the helicase domain of the latter were suggested to be pro-oncogenic in a transgenic Eμ-*Myc* mouse model [101]. Notably, in DLBCL, but also in kidney renal clear cell carcinoma (KIRC), high *ZRANB3* expression levels correlate with better prognosis (Figure 2C and Supplementary Figure S2 showing recurrence-free survival). Instead, the association of high *ZRANB3* expression with worse prognosis in lung adenocarcinoma (LUAD) may reflect the deleterious effects of certain functional mutations as a strategy used by the tumour to sustain abnormal proliferation (Figure 2C, Supplementary Figure S2 and Table S2).

Nevertheless, *ZRANB3* can be also downregulated, an effect that can be more easily interpreted in light of its tumour-suppressor function (Figure 2B). Downregulation is observed in adrenocortical carcinoma (ACC), kidney chromophobe (KICH), ovarian cancer (OV), testicular germ cell tumour (TGCT), and thyroid carcinoma (THCA), with a stronger variation in acute myeloid leukaemia (LAML) (Figure 2B). However, in ACC and KICH, low HLTF expression correlates unexpectedly with worse prognosis, suggesting a different scenario (Figure 2D, Supplementary Figure S2). Moreover, the opposite outcomes observed in different kidney cancer subtypes, which differ for their immune-infiltrative traits and specific biomarkers expression, suggest a tissue-specific tumour regulatory function for ZRANB3 [102,103]. Undoubtedly, the spectrum of ZRANB3-associated survival supports its potential as a novel prognostic biomarker.

At the protein level, ZRANB3 mutations occur at relatively low frequency compared to other alterations, as they are present only in some cancers and in less than 10% of patients overall and can correlate with moderate either up- or downregulation of *ZRANB3* mRNA (Figure 2A, Supplementary Figure S3A). Mutations are more frequent in endometrial cancer (EC) and melanoma (MC), and overall can be amplified or deleted depending on the presence of certain CNAs (Supplementary Table S2). This association between ZRANB3 functional mutations and the presence of CNAs can be particularly observed in the PIP and APIM domains [79,80]. Also, *ZRANB3* CNAs correlate with abnormal mRNA expression overall (Supplementary Figure S3B). This genetic buffering may either promote tumour progression or be an alternative way of the cell to rescue normal conditions [104,105]. Therefore, careful evaluation in each tumour type and subtype is needed.

### 4.2. Correlation of ZRANB3 Expression with TP53 Across TCGA Cancers

How might this role of ZRANB3 be connected to p53 function in cancer? To address this question, we analysed the correlation between *ZRANB3* and *TP53* expression across all TCGA cancer types considering (i) the *TP53* status, wt or mutated, and (ii) its association with cell proliferation as function of *MKI-67* expression, which also becomes downregulated by p53 [96]. The reader can find in the supplementary section the methods used for data analysis and the full data outcomes (Supplementary Information, Supplementary Tables S3 and S4).

**Figure 3:**
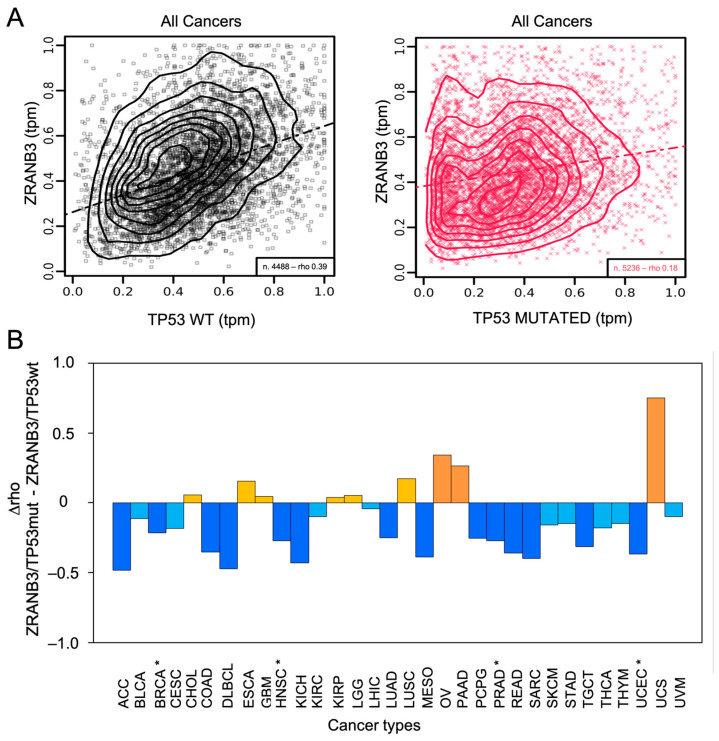
(**A**) Co-expression of *ZRANB3* and *TP53* in all TCGA cancer data; *n*: number of samples; rho: Pearson’s correlation coefficient; tpm: transcript per million; dotted-dashed lines are linear regression lines. (**B**) Effects of *TP53* mutations on the correlation between *ZRANB3* and *TP53* across all TCGA cancer types [18]. The difference between Pearson’s correlation coefficients (D rho: rho_ZRANB3/TP53mut_-rho_ZRANB3/TP53wt_) are shown (full details are reported in Supplementary Table S3). Cancers with D rho > 0.2 are coloured in orange, while cancers with 0 < D rho < 0.2 are coloured in yellow (increased correlations). Cancers with D rho < −0.2 are coloured in blue, while cancers with −0.2 < D rho < 0 are coloured in light blue (decreased correlations). Statistically significant cancers are marked (*).

We found that the mRNA expression patterns of *ZRANB3* and *TP53* are moderately correlated across cancers overall, but that this correlation is substantially reduced when *TP53* is mutated (rho_ZRANB3/p53wt_ = 0.39; rho_ZRANB3/p53mut_ = 0.18) (Figure 3A and Supplementary Table S3). This effect is even more evident when examining individual cancer types (Figure 3B and Supplementary Table S3). Particularly, the correlation is statistically significant in breast cancer (BRCA: rho_ZRANB3/p53wt_ = 0.34; rho_ZRANB3/p53mut_ = 0.12), where mutations in *TP53* have a predominant role in promoting abnormal cell proliferation [106,107], but also in head and neck squamous cell carcinoma (HNSC: rho_ZRANB3/p53wt_ = 0.57; rho_ZRANB3/p53mut_ = 0.3), prostate adenocarcinoma (PRAD: rho_ZRANB3/p53wt_ = 0.49 and rho_ZRANB3/p53mut_ = 0.22), and uterine corpus endometrial carcinoma (UCEC: rho_ZRANB3/p53wt_ = 0.59 and rho_ZRANB3/p53mut_ = 0.23) [108] (Supplementary Figure S4—coexpression panels of ZRANB3 and TP53 in these tumours). Unfortunately, among the main BRCA subtypes, only Luminal A and Luminal B have significant data, which support the overall result (Supplementary Figure S5—coexpression panels of ZRANB3 and TP53 in these BRCA subtypes).

Also, the more complex correlation patterns as a function of low or high proliferation rates (low or high *MKI-67* expression) observed in BRCA, HNSC, PRAD, and UCEC cannot be fully interpreted yet due to the limited statistical power (Supplementary Figure S6). Instead, what emerges significantly from a more fine-tuned analysis of such data is that all of these cancers show a negative correlation between *ZRANB3* and the two-way interaction between *TP53* and *MKI-67* (Supplementary Table S4—multivariate correlation of *ZRANB3* with *TP53* and *MK-I67*). Specifically, in patients with low *MKI-67* expression, *ZRANB3* expression increases as *TP53* expression increases, while in patients with high *MKI-67* expression, *ZRANB3* expression decreases as *TP53* expression increases. This observation, which can also be found for pheochromocytoma and paraganglioma (PCPG), is notably consistent with previous results and supports the idea that ZRANB3 and p53 may functionally diverge under high-proliferation conditions [96].

Since both the limited sample size and the marked heterogeneity of TCGA cohorts could contribute to outcome variability, an in-depth evaluation of the data based, for instance, on patient characteristics, tumour subtypes, and diverse treatments could better highlight complex details.

Altogether these data support at an inferential level the existence of a ZRANB3/p53 axis, which becomes activated in early tumour stages as a cellular strategy to counteract tumour development.

## 5. Conclusions

Biomarkers are key targets in personalised cancer therapy. Determining the unique biomarker profile of an individual’s tumour could help tailor more effective treatments, improve outcomes, and reduce side effects, thus ultimately enhancing overall patient care. Therefore, it is essential to discover and characterise biomarkers, study their structural and functional properties in depth, and elucidate their molecular pathways within the cellular milieu.

ZRANB3 is member of the SNF2-family of chromatin remodelers with an ancient origin. It uniquely combines ATP-dependent motor remodeling action with a nuclease function, primarily in the context of genome stability and in cooperation with other partners. For this role and for its association with p53 during damage repair, ZRANB3 is considered a tumour suppressor, although its involvement in DNA synthesis and cell metabolism may indicate alternative context-dependent functions influenced by the oncogenic background, replication stress, and p53 status. Indeed, the correlation between *ZRANB3* and *TP53* is observed across tumours overall, but it is modulated by *TP53* mutations and high proliferation rates, ultimately supporting the hypothesis of a functional ZRANB3/p53 axis in cancer.

## Figures and Tables

**Table 1:** List of DNA and protein (PCNA) substrates used to test ZRANB3 activity. For each assay and substrate, the effects on protein function (→) of the correspondent ZRANB3 and ZRANB3 domain and the mutants tested are reported.

Functional Assay	Substrate	ZRANB3 Construct → Observed Effect	Reference
Nuclease ATP-ase	Splayed arms	SRD SRD (L760A, D761A, I762A) → abolished ATP-ase and nuclease activities SRD (W790A, S791A, S792A) → abolished ATP-ase activity Δ651–720 → no effect on ATP-ase activity ΔNZF → no effect on ATP-ase activity ΔAPIM → no effect on ATP-ase activity ΔHNH → moderately decreased ATP-ase activity and abolished nuclease activity	[62] Badu-Nkansah et al. 2016
Fork remodeling	Model fork	ATP-ase + SRD (only) → retained fork remodeling as the FL	[62] Badu-Nkansah et al. 2016
Nuclease ATP-ase	Splayed arms	ZRANB3 FL Cancer associated (T66A, R169H, G401D) → abolished ATP-ase and nuclease activities Cancer associated (R947Q) → reduced ATP-ase and nuclease activities Cancer associated (R947*, D1020Y) → reduced ATP-ase activity and abolished nuclease activity HNH (Δ975–1013 and Δ972–1010) → loss of endonuclease activity and reduction of ATP-ase activity HNH (K984A, R988A, K988A) → no effect HNH (K997A, R1009A) → reduced ATP-ase and nuclease activities HNH (K1046A+R1048A) → no effect on ATP-ase activity, reduced nuclease activity HNH (H1015A) → no effect HNH (D1020A, H1021A, H1045A) → no effect on ATP-ase activity, abolished nuclease activity HNH (N1036A) → no effect on ATP-ase activity, reduced nuclease activity	[27] Sebesta et al. 2017
Protein interaction	PCNA	PIP PIP (Q519A, F525A, F526A) → abrogated protein interaction	[27] Sebesta et al. 2017
Immunoprecipitation (IP)	PCNA	ZRANB3 FL PIP (Q519A, F525A, F526A) → abrogated protein interaction	[54] Weston et al. 2012
Anti-ubiquitin western blotting	K63_polyUb PCNA	ZRANB3 FL NZF (W625A, Y632A, N634A) → abolished interaction NZF (T631A) → reduced interaction NZF (I633A, E642A) → no effect NZF (M643A) → compromised affinity for oligomeric Ub but retained interactions with K63_polyUb	[54] Weston et al. 2012
Recruitment at DNA damage site	PCNA	PIP PIP long mutant (from 519 to 526) → defective recruitment APIM ΔAPIM → defective recruitment APIM (F1073A) → defective recruitment	[79] Ciccia et al. 2012

**Table 2:** ZRANB3 PPI network retrieved from the STRING database [84], including both experimentally proven (physical interaction) and putative (co-expressed or with similar function) partners within different cell cycle regulatory mechanisms.

Physical Interaction	
PCNA	Proliferating cell nuclear antigen, auxiliary protein of Pold and Pole: involved in DNA replication, repair, and DDT
**Co-Expression**	
CEP76	Centrosomal protein of 76 KDa: regulation of centriole duplication
GST_CD	Glutathione S-transferase C-terminal domain-containing protein: may be linked with cell viability and apoptosis in bronchial epithelial cells
CEP44	Centrosomal protein of 76 KDa: maintains centrosome cohesion by interacting and stabilizing the CROCC complex
**Similar Function or Same Pathways**	
SMARCAL1	SWI/SNF-related matrix-associated actin-dependent regulator of chromatin subfamily A-like protein 1: catalyses the rewinding of ssDNA bubbles and protects stalled replication forks
DNA2	DNA replication ATP-dependent helicase/nuclease DNA2: involved in Okazaki fragment processing and resection of 5′-end during DSB repair
WRN	Werner syndrome ATP-dependent helicase and 3′–5′ exonuclease with preference for 5′-overhang dsDNs: may be critical for HR
HLTF	Helicase-like transcription factor with ATP-dependent nucleosome-remodeling activity: involved in DNA repair in general and transcriptional control
RAD51	DNA repair protein RAD51 homologue 1: critical during HR for the formation of a joint molecule between a processed DNA break and the repair template
BRCA2	Breast cancer type 2-associated protein: involved in DSB repair and/or HR
ABRO1	BRISC complex subunit Abro1: involved in inhibition of DNA2 nuclease/WRN helicase-mediated degradation of stalled forks
MUS81	Crossover junction endonuclease MUS81 with preference for 3′-flap structures, replication forks, and nicked Holliday junctions
MRE11	Both single-strand endonuclease and double-strand-specific 3′–5′ exonuclease are involved in the repair of DSBs via homologous recombination (HR)
FBH1	F-box DNA helicase 1 and 3′–5′ DNA helicase component of the SCF(FBH1) E3 ubiquitin ligase complex: prevents extensive strand exchange during HR
FANCD2	Fanconi anaemia group D2 protein: required for the repair of DSBs, both by HR and single-strand annealing
FANCM	Fanconi anaemia group M protein, component of the Fanconi anaemia (FA) core complex: activation of FA pathway, leading to monoubiquitinated FANCI-FANCD2 complex in response to DNA damage
RECQ1	ATP-dependent DNA helicase Q1: unwinding dsDNA in 3′–5′ direction
RECQ5	ATP-dependent DNA helicase Q5: critical for DNA replication, transcription, and repair
BLM	3′–5′ ATP-dependent DNA helicase: participating in the 5′-end resection of DNA during DSB repair

## Data Availability

No new data were created or analyzed in this study. Data sharing is not applicable to this article.

## Supplementary Materials

The following supporting information can be downloaded at: https://www.mdpi.com/article/10.3390/ijms27020574/s1. References [36,39,40,41,42,43,44,45,46,60,66,67,68,69,71,72,73,97,98,99] are cited in the Supplementary Materials.
