# Supplementary material for: The Zinc Finger Ran-Binding Protein 3 (ZRANB3): An Advanced Perspective
Source: Int J Mol Sci. 2026 Jan 6;27(2):574. doi: 10.3390/ijms27020574 (PMC12841045; doi:10.3390/ijms27020574)
Supplement: Supplementary file 1 [file ijms-27-00574-s001.zip › ijms-4042297-supplementary.pdf]

Supplementary Material

The Zinc-finger Ran Binding protein 3 (ZRANB3): an advanced perspective

Paride Pelucchi [1#], Ettore Mosca [1#], Nika Tomsič [2], Yossma Waheed [2], Wendalina Tigani [3], Alice Chiodi [1], Aditya Mojumdar [4], Marco Gerdol [3*], and Matteo De March [2*]

[1] Istituto di Tecnologie Biomediche (ITB), Consilio Nazionale delle Ricerche (CNR), via F.lli Cervi 93, Segrate, Milano, Italy
[2] Laboratory for Environmental and Life Sciences, University of Nova Gorica, Vipaska 13, SI-5000, Nova Gorica, Slovenia
[3] Department of Life Science, University of Trieste, via Licio Giorgieri 5, 34100, Trieste, Italy
[4] Department of Biochemistry and Microbiology, University of Victoria, BC V8W 2Y2, Victoria, Canada

This file contains Supplementary Material and Methods with appropriate references, Supplementary Figures S1-S6, and Supplementary Tables S1-S4

Phylogenetic analyses

The presence of ZRANB3 orthologs was evaluated across Metazoa using a combination of homology searches carried out with BLASTp [1] (using the human protein sequence Q5FWF4 as a query, with an e-value threshold of 1e-5), the assessment of the presence of the expected conserved domains through Interproscan v.5 [2], and the evaluation of the distribution of homologous sequences in fully sequenced genomes though the Ensembl portal [3]. Homology searches were further extended to non-metazoan organisms, combining the aforementioned strategies with the identification of distantly related sequences displaying the same domain architecture with CD-search [4].

The complete ZRANB3 protein sequences from a selection of species representative of animal biodiversity (besides human, *Anolis carolinensis*, *Acipenser ruthenus*, *Bolinopsis microptera*, *Branchiostoma belcheri*, *Bufo bufo*, *Corticium candelabrum*, *Callorhinchus milii*, *Mus musculus*, *Nematostella vectensis*, *Ornithorhynchus anatinus*, *Patiria miniata*, *Petromyzon marinus* and *Saccoglossus kowalevskii*) were recovered from the non-redundant protein database, together with those of the choanoflagellates *Monosiga brevicollis* and *Salpingoeca rosetta* (**Figure S1A**).

Similarly, SMARCAL1 orthologous sequences, identified using the same approach outlined above, were obtained from the same species. Finally, the proteins encoded by human paralogous genes (CHD1, CHD2, CHD3, CHD4, CHD4, CHD6, CHD7, CHD8, CHD9, ERCC6, SMARCA2, /SMARCA4 and /SMARCAD1) were also added to the sequence collection (**Figure S1A**).

All retrieved protein entries were subjected to multiple sequence alignment (MSA) with MUSCLE [5]), which was subsequently trimmed to only keep the homologous regions shared by all proteins (i.e. the DEXDc and HELIc domains) and further processed to remove sites affected by significant gaps (i.e. missing in >50% of the aligned sequences). The resulting manually curated MSA was used as an input for a Maximum Likelihood phylogenetic inference analysis, which was carried out with IQ-TREE [6], according to a LG+I+G4 model of molecular evolution, which was selected by ModelFinder [7] as the best fitting one for the dataset based on the Bayesian Information Criterion [8]. The reliability of tree topology (**Figure S1A**) was evaluated with 1000 ultrafast bootstrap replicates [9].

Homology-based dsDNA binding site analysis

To define the boundaries of the ZRANB3 ATP-ase motor-remodeling domain (ATP-ase RD), we considered the sequence and structural features of ATP-ase core plus the HD1 and HD2 regions [10]. The AlphaFold model of ZRANB3 ATP-ase RD [11] was used as query for a DALI search [12] against the Protein Data Bank (PDB) [13]. The list of >1000 matches were filtered considering together z score >10% and identity value >15%. Apo-structures were excluded, as well as those in complex with ssDNA or RNA, and those lacking either the N- or the C-terminal RD. The target structures were analysed using PISA [14] to get the experimental nucleic acid binding residues considering those interactions within 4.5Å. Next, we used PyMol [15] to i) overlap separately both ATP-ase RD subunits of ZRANB3 to the correspondent lobes of each target structure, ii) retrieve the ZRANB3-DNA binding residues (within 4.5Å) and iii) compare the potential functional matches on the same DNA binding sites (R, K, N, Q, E, H, F, Y, T) [16] that were retained (conserved) or eventually substituted (gain vs not present) on ZRANB3 ATPase RD with respect to the target. To get more reliable statistics on each putative DNA binding sites, we also used HybridDBRpred [17]. Mutations were checked at cBIO portal for Cancer genomics [18]. **Figure S1B and S1C** report all putative ZRANB3 ATP-ase RD - DNA binding residues. The most significant are those with a combination of at least 2 out of the four criteria i) homology-based DNA binding probability (%>50), ii) HDBRpred score > 50, iii) presence/absence of mutation, and iv) with/without loss of function.

TCGA data analysis

The publicity available *ZRANB3* alterome was inspected at the at cBIO portal for Cancer genomics and TCGA [18, 19] (**Figure 2A-2B, Figure S3**). Kaplan-Mayer plots of overall survival were retrieved from GEPIA2 [20] and stratified on the ZRANB3 mRNA expression median (**Figure 2C, Figure S2**). This represents a more standard approach especially when rarer tumours, such as KICH, which contain less amount of data, are

considered. Stratification on the 1st (tumours with higher ZRANB3 expression) and 4th (the lower ZRANB3 expressing tumours) quartiles, thus excluding 50% of the samples expressing an average amount of ZRANB3, gave comparable results with similar statistical significance [20].
Gene expression data (TPM, transcript-per-million) for *ZRANB3*, *TP53* and *MKI67* were downloaded either by means of the R package (**Figure 3, Figures S4-S6**). The list of subjects with mutations on *TP53* was obtained from the Genomic Data Commons (GDC) portal [21]. In each tumour, subjects with *ZRANB3* or *TP53* expression lower than the $Q_1 - 1.5\ (Q_3 - Q_1)$ or higher than $Q_3 + 1.5\ (Q_3 - Q_1)$ were not considered, where Q_1 and Q_3 denote the first and third quartiles, respectively. We used linear regression model to fit these data, while the significance of each Fisher's test on ZRANB3/TP53 correlation was attributed for p-values < 0.05 (**Table S3**). The same procedure was applied considering low- and high-expression values of *MKI-67* (**Figure S6**). Instead, to assess the statistical interaction (correlation) among *ZRANB3*, *TP53* and *MKI-67*, we used the multivariate model:

$$\mathbf{y} = b_0 + b_1\mathbf{x}_1 + b_2\mathbf{x}_2 + b_3\mathbf{x}_3 + b_4\mathbf{x}_1\mathbf{x}_2 + b_5\mathbf{x}_1\mathbf{x}_3 + b_6\mathbf{x}_2\mathbf{x}_3 + \boldsymbol{\varepsilon}$$

with y: *ZRANB3* expression (tpm), x1: *TP53* expression (tpm), x2: *TP53* mutational state, x3: *MKI-67* expression (tpm) (**Table S4**).

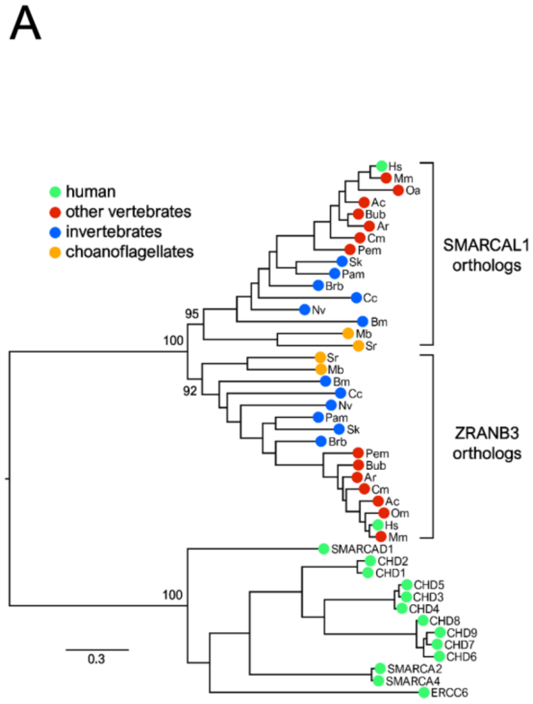

B

ZRANB3 ATP-ase_NRD dsDNA binding residues	Functional effect with respect to the same DNA binding site in target structures	Homology-based ZRANB3 DNA binding probability (%)	HybridDBR Pred score	Cancer mutations
R91	GAIN (mostly sobstituted with L or M)	64.3	0.17	
Q114	CONSERVED (mostly sobstituted with E, H, K)	60.7	0.11	Bladder (Q114*_del)
N115	GAIN (mostly sobstituted with G)	67.9	0.10	
K116	GAIN (mostly sobstituted with S or D)	78.6	0.11	
T117	GAIN/CONSERVED (mostly sobstituted with P or K)	39.3	0.10	
*Y132**	CONSERVED (mostly sobstituted with Y)	25.0	0.61	Kidney Renal (Y132D)
T136	GAIN (mostly sobstituted with I or L)	75.0	0.11	
H160*	CONSERVED (mostly sobstituted with H)	32.1	0.76	
Y161*	CONSERVED (mostly sobstituted with R)	64.3	0.76	
K163*	CONSERVED (mostly sobstituted with K)	92.9	0.75	
R165*	GAIN (sobstituted with A, F, V or G)	14.3	0.72	
N166*	CONSERVED (mostly sobstituted with Q, E or K)	64.3	0.73	
T168*	CONSERVED (mostly sobstituted with K, E or T)	78.6	0.53	
R169	GAIN (mostly sobstituted with I or L)	92.9	0.45	Endometrial (R169H. R169C)
R194	CONSERVED (mostly sobstituted with N)	78.6	0.10	
E196	CONSERVED (sobstituted with E, K or Y)	10.7	0.10	Lung Squamous Cell (E196D); Head and Neck (E196*_del)
E197	CONSERVED (mostly E)	67.9	0.08	Lung Adenocarcinoma (E197K)
T214	CONSERVED (sobstituted with E)	3.6	0.28	
K218	CONSERVED (sobstituted with R)	3.6	0.18	
N222	not present	3.6	0.15	
H224	CONSERVED (sobstituted with K, F or E)	53.6	0.12	
R226	not present	78.6	0.10	Colorectal (X226_splice)
Y227	not present	75.0	0.10	
F228	not present	75.0	0.11	
K230	not present	50.0	0.10	
R231	not present	75.0	0.11	
Q233	not present	78.6	0.10	
R237	GAIN/CONSERVED (sobstituted with A, K or G)	82.1	0.13	Bladder (R237K)

C

ZRANB3 ATP-ase_CRD dsDNA binding residues	Functional effect with respect to the same DNA binding site in target structures	Homology-based ZRANB3 DNA binding probability (%)	HybridDBR Pred score	Cancer mutations
T287	not present	3.6	0.06	
F289	GAIN/CONSERVED (sobstituted with R or not present)	7.1	0.06	
E291	GAIN/CONSERVED (sobstituted with L or N)	28.6	0.06	Bladder (E291Q)
E293	CONSERVED (sobstituted with K)	3.6	0.07	
K294	GAIN/CONSERVED (sobstituted with K or not present)	14.3	0.08	
R297	not present	7.1	0.07	Cervical (R297K)
T298	not present	25.0	0.06	
N300	mostly not present	71.4	0.09	
E305	GAIN (sobstituted with M, V or not present)	85.7	0.09	
T306	GAIN/CONSERVED (sobstituted with G, T or not present)	78.6	0.09	
T312	GAIN (mostly sobstituted with M)	85.7	0.10	
R313	CONSERVED (mostly sobstituted with F or Q)	35.7	0.09	Endometrial (R313C; R313H)
F315	not present	10.7	0.10	
K316	CONSERVED (mostly sobstituted with K or R)	78.6	0.09	
Q317	not present	3.6	0.10	
H346	CONSERVED (mostly sobstituted with Q)	89.3	0.19	
H347	GAIN (mostly sobstituted with M)	82.1	0.17	
E376	GAIN (sobstituted with S)	3.6	0.28	
R377	CONSERVED (mostly sobstituted with R)	89.3	0.13	
Q398	CONSERVED (mostly sobstituted with R)	82.1	0.27	
Q402	CONSERVED (sobstituted with E, Y, T)	10.7	0.25	Sarcoma (X402_splice)
Y418	GAIN (mostly sobstituted with D)	71.4	0.1	
H423	CONSERVED (mostly sobstituted with T or Q)	67.9	0.12	
T453	CONSERVED (sobstituted with T)	3.6	0.08	
R461	CONSERVED (sobstituted with R)	3.6	0.15	Endometrial (R461L); Cervical (R461H)
K462	CONSERVED (mostly K)	82.1	0.09	

Figure S1. A. Maximum likelihood phylogeny of ZRANB3 and other evolutionarily related SNF2-family helicases, based on the multiple sequence alignment of the helicase domains. Only the bootstrap support values of the main nodes of the tree are shown. Ac: *Anolis carolinensis*; Ar: *Acipenser ruthenus*; Bm: *Bolinopsis microptera*; Brb: *Branchiostoma belcheri*; Bub: *Bufo bufo*; Cc: *Corticium candelabrum*; Cm: *Callorhinchus milii*; Hs: *Homo sapiens*; Mb: *Monosiga brevicollis*; Mm: *Mus musculus*; Nv: *Nematostella vectensis*; Oa: *Ornithorhynchus anatinus*; Pam: *Patiria miniata*; Pem: *Petromyzon marinus*; Sk: *Saccoglossus kowalevskii*; Sg: *Salpingoeca rosetta*. **B.** List of all putative *in-silico*

ZRANB3-DNA binding residue found by homology modeling on N-terminal ATP-ase_RD (dark green) with their functional effect (gain, conserved or not present with respect to the same binding site in target structures), homology-based binding probability (expressed as the relative abundance in target structures %), HybridDBRpred score [17] and mutations. Residues in bold with % >50, residues with * with Pred score > 50, residues in italic with significative loss of function mutations. Arrows indicate the most significant. **C.** Same information as in panel B for the C-terminal ATP-ase RD (light green).

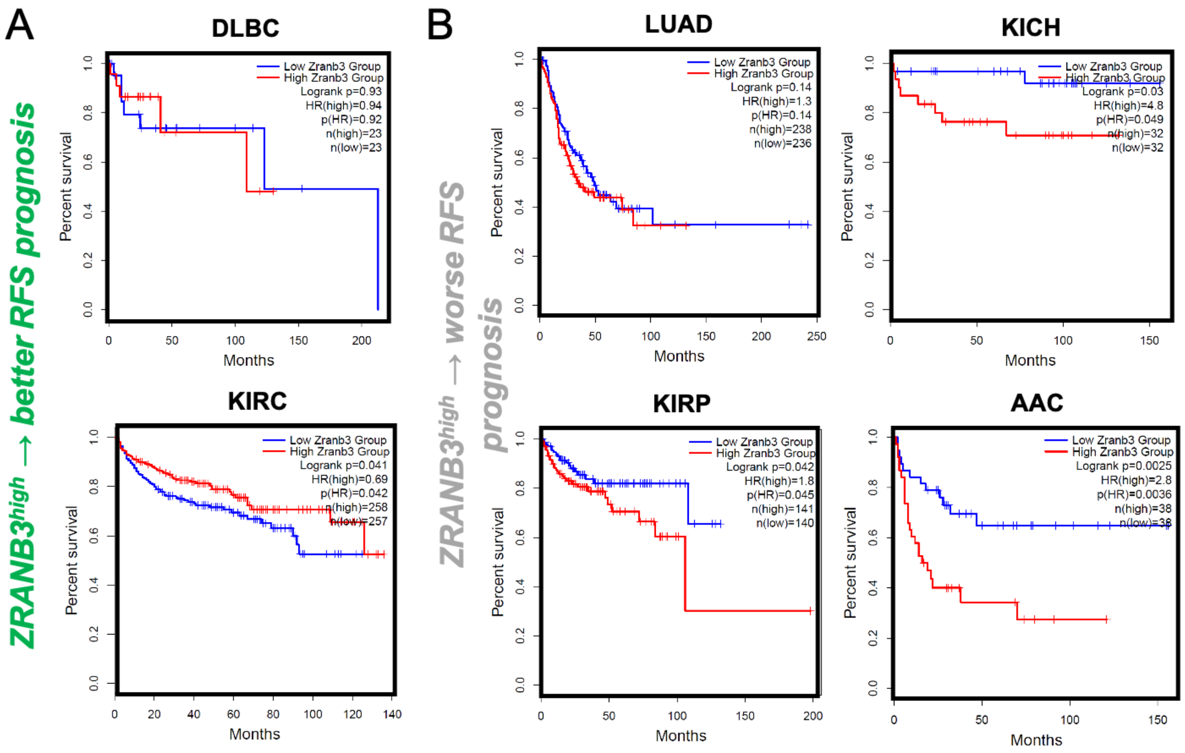

Figure S2. **A.** Kaplan-Meier representations of Recurrence-Free Survival (RFS) of tumour patients with high (red) and low (blue) ZRANB3 mRNA expression [20]. For each tumour type the patients' cohorts are divided into two equal sub-groups at the median of ZRANB3 expression. Each plot shows the number of patients in each sub-groups, the Hazard Ratio (HR) representing the difference of risk between the two groups (>1 if the risk is higher in the high-expressing group and <1 if the risk is lower), and the p value resulted from log rank test associated (logrank p). DLBC: diffuse large B-cell lymphoma. KIRC: kidney renal clear-cell carcinoma; **B.** Same plot as in panel C for lung adenocarcinoma (LUAD), kidney chromophobe (KICH), kidney renal papillary-cell carcinoma (KIRP), and adrenocortical carcinoma (ACC).

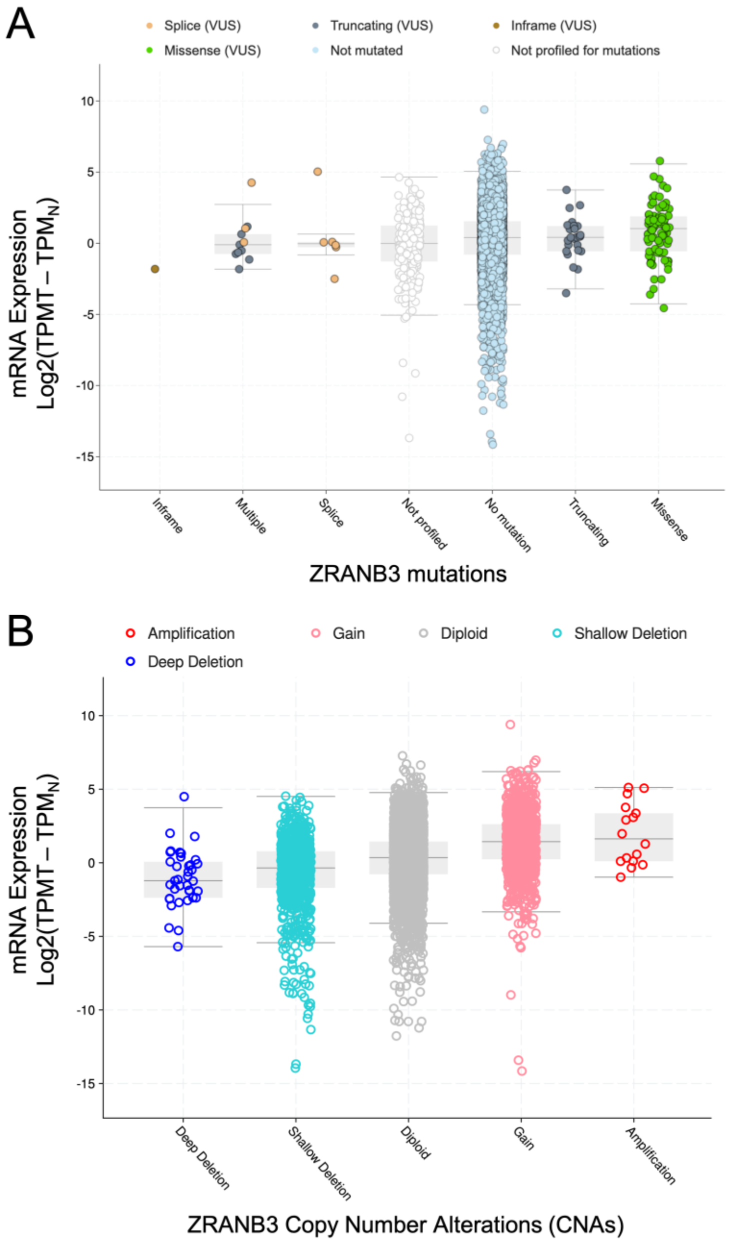
A
Splice (VUS)
Truncating (VUS)
Inframe (VUS)
Missense (VUS)
Not mutated
Not profiled for mutations
mRNA Expression
Log2(TPMT – TPMN)
10
5
0
-5
-10
-15
Inframe
Multiple
Splice
Not profiled
No mutation
Truncating
Missense
ZRANB3 mutations
B
Amplification
Gain
Diploid
Shallow Deletion
Deep Deletion
mRNA Expression
Log2(TPMT – TPMN)
10
5
0
-5
-10
-15
Deep Deletion
Shallow Deletion
Diploid
Gain
Amplification
ZRANB3 Copy Number Alterations (CNAs)

Figure S3. A. ZRANB3 mRNA expression pattern (difference between tumour and normal) versus mutation type (splice, truncating, in-frame, and missense) across all TCGA tumours [18]. **B.** ZRANB3 mRNA expression pattern (difference between tumour and normal) versus Copy Number Alterations (CNAs): Deep Deletions (deletions of large portion of the gene), Shallow Deletion (deletions of a small portion of the gene), Diploid, Gain (amplification of few copies of the gene), and Amplification (strong amplification of the gene copies) across all TCGA tumours [18].

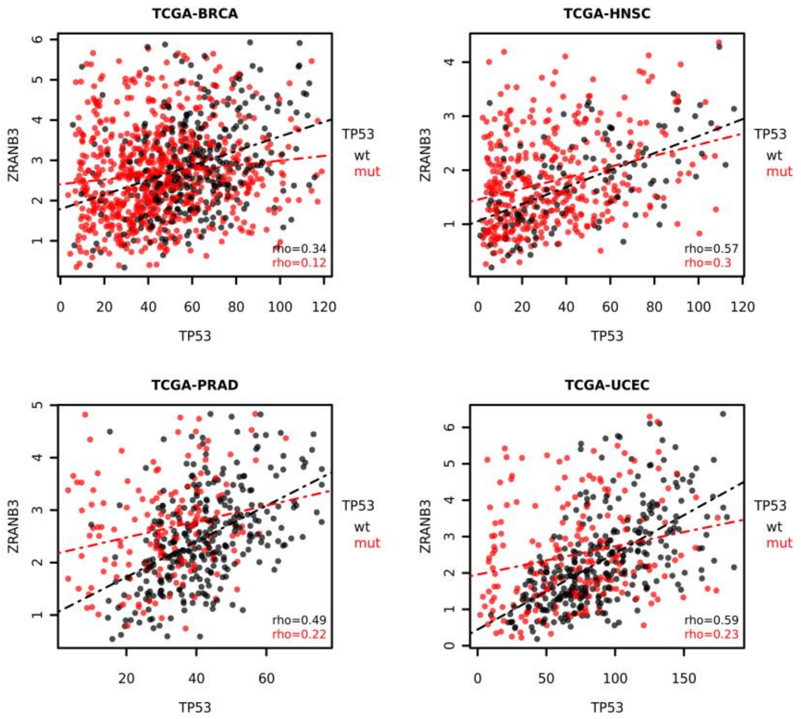

Figure S4. A. Co-expression of ZRANB3 and TP53 for the statistically significant tumours of Table S3. *TP53* WT (black dots); *TP53* MUT (red dots). tpm: transcript per million; dot-dash lines are linear regression lines.

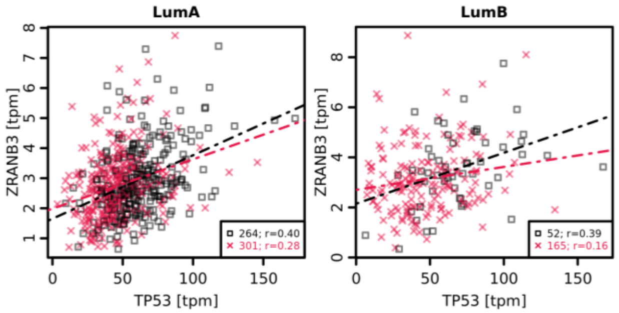

Figure S5. Co-expression of *ZRANB3* and *TP53* in the statistically significant BRCA subtypes Luminal A and Luminal B [21] (Table S3). *TP53* WT (black squares); *TP53* MUT (red crosses). r: Pearson' correlation coefficient; tpm: transcript per million; dot-dash lines are linear regression lines.

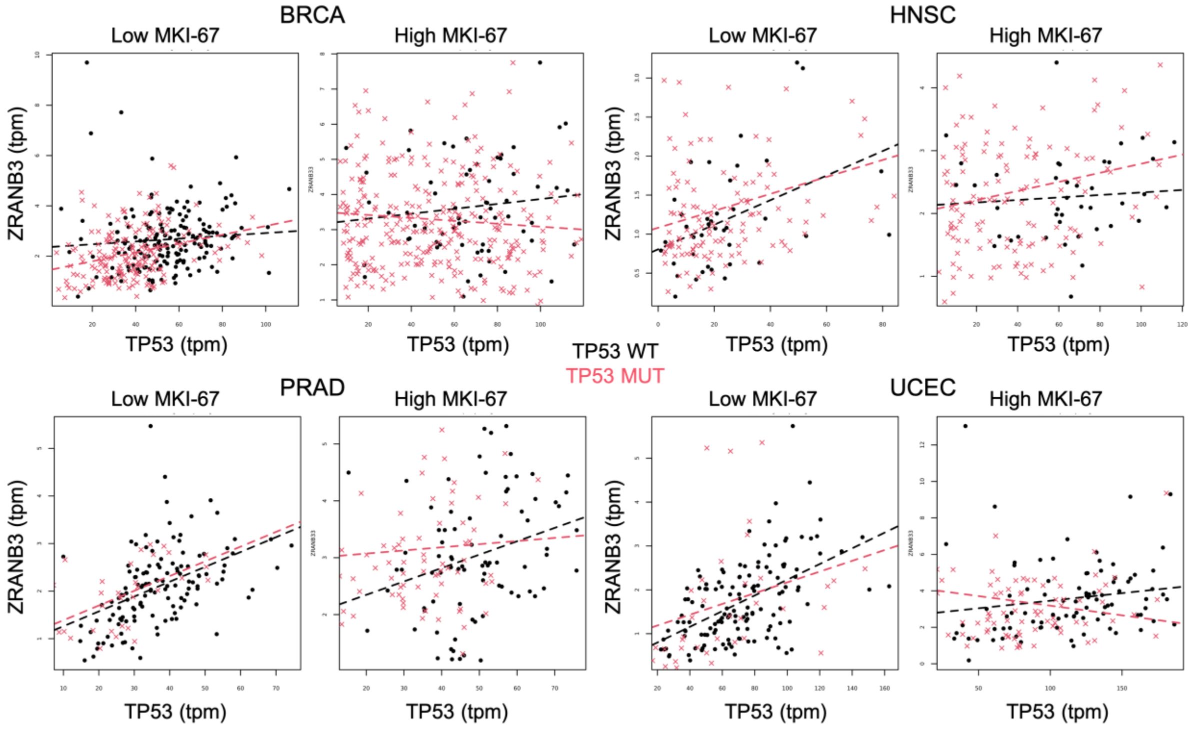

Figure S6. Co-expression of *ZRANB3* and *TP53* as function of low- and high- *MKI-67* expression for the statistically statistically significant tumours (Supplementary Tables S3–S4). *TP53* WT (black dots); *TP53* MUT (red crosses). tpm: transcript per million; dot-dash lines are linear regression lines. In BRCA: at low *MKI-67* rho$_{ZRANB3/TP53wt}$ is 0.082 (p-value 0.283), while rho$_{ZRANB3/TP53mut}$ is 0.305 (p-value 0.000); at high *MKI-67* rho$_{ZRANB3/TP53wt}$ is 0.130 (p-value 0.307), while rho$_{ZRANB3/TP53mut}$ is -0.077 (p-value 0.184). In HNSC: at low *MKI-67* rho$_{ZRANB3/TP53wt}$ is 0.429 (p-value 0.006), while rho$_{ZRANB3/TP53mut}$ is 0.291 (p-value 0.001); at high *MKI-67* rho$_{ZRANB3/TP53wt}$ is 0.082 (p-value 0.608), while rho$_{ZRANB3/TP53mut}$ is 0.197 (p-value 0.024). In PRAD: at low *MKI-67* rho$_{ZRANB3/TP53wt}$ is 0.463 (p-value 0.000), while rho$_{ZRANB3/TP53mut}$ is 0.534 (p-value 0.002); at high *MKI-67* rho$_{ZRANB3/TP53wt}$ is 0.289 (p-value 0.009), while rho$_{ZRANB3/TP53mut}$ is 0.077 (p-value 0.490). In UCEC: at low *MKI-67* rho$_{ZRANB3/TP53wt}$ is 0.539 (p-value 0.000), while rho$_{ZRANB3/TP53mut}$ is 0.351 (p-value 0.019); at high *MKI-67* rho$_{ZRANB3/TP53wt}$ is 0.165 (p-value 0.124), while rho$_{ZRANB3/TP53mut}$ is -0.1891 (p-value 0.065).

	ZRANB3	SMARCAL1	WRN	DNA2
**Functional domains**	ATP-ase helicase-like: dsDNA translocation and remodeling<br>PIP: PCNA binding<br>NZF: recruitment by Ub-PCNA<br>SRD: Substrate specificity<br>HNH: nuclease<br>APIM: PCNA binding	RBD: binding to RPA<br>HARP1/2: DNA annealing<br>ATP-ase helicase-like: dsDNA translocation and remodeling	ExoND: nuclease<br>ATP-ase helicase + ZF: DNA unwinding<br>RQC: primarily binding to DNA<br>HRDC: protein interaction	ssDBD: binding to ssDNA<br>ND: nuclease<br>β-barrel: DNA binding<br>ATP-ase helicase: DNA unwinding
**ATP-ase activity**	Dependent on DNA binding	Dependent on DNA binding	Dependent on DNA binding	Weak and linked to helicase function
**Helicase activity**	None	annealing	3’→5’ unwinding	5’→3’ unwinding
**Exonuclease activity**	None	None	3’→5’	ATP-dependent Flap Endo/Exonuclease
**Endonuclease activity**	Structure-specific by HNH (5’-flap)	None	5’-end (fragment 70-270)	5’-end
**DNA binding specificity**	Branched DNAs<br>Splayed arm, ssDNA/dsDNA junctions, 3-way junctions	Branched DNAs<br>Splayed arm ssDNA/dsDNA junction, 3-way DNA junctions, 4-way Holliday junctions, dsDNA with internal ssDNA gaps	ds DNAs<br>D-loops<br>Holliday junctions, replication forks, recombination intermediates, repair intermediates (13, 18), telomeric ends, forked duplexes, 5′-overhang duplexes	ss DNAs<br>Flap structures<br>Okazaki fragments
**DNA:RNA hybrid removal**	R-loops	R-loops	R-loops	R-loops

**G-quadruplex resolution**	Yes	Yes	Yes	Yes
**Role in genome stability**	RFR, TLS, DSB, HR	RFR, ALT	HR, NHEJ, BER, Telomere stability	DSB, Telomere stability, HR, Okazaki fragments processing

Table S1. Summary of the main structural and functional properties known so far for ZRANB3, SMARCAL1, WRN and DNA2 (RFR: replication fork reversal; TLS: Translesion Synthesis; DSB: double strand break; HR: homologous recombination; ALT: alternative lengthening of telomeres, NHEJ: non-homologous end joining; BER: base excision repair).

Cancer Type	Mutations (CNVs)						
	**ATP-ase N/C RD**	**PIP-box**	**NZF**	**SRD**	**HNH**	**APIM**	**none**
Thyroid Cancer	V440A; **G403***						
Adrenocortical Carcinoma							**T593R (Gain)**; T891I
Ovarian Epithelial Tumor							**E515K**
Glioblastoma					L1040P; **X1048_splice**		S930Y (Gain)
Sarcoma	A393P (Shallow del); **X402_splice**						
Bladder Cancer	**D157H (Shallow del)**; D138N (Gain); M296I; E291Q; **Q114* (Shallow del)**; M296I; R237K; **X283_splice**				R1009K (Gain); S997L		**D500V (Gain)**; **D23H (Amp)**; L872V
Hepatobiliary Cancer	**R364C (Deep del)**; I295L			S756N (Gain)			**Q716H**
Prostate Cancer							**P594S**
Renal Clear Cell Carcinoma	Y132D			V839L			
Endometrial Cancer	**R461L**; P195S; **W456***; F414C; **R313C**; T66A; **K340T**; R169H; G401D; **E97***; **R169C**; A393V (Gain); R313H; **E79***; **X54_splice**; A137T; V119D; **I295***; A239V; L404V; **R430***; F384C	**R523***		**R790***; G852C; **R855S**; A797V; M735I	**R947***; **C1041Hfs*13**; **E959A**; **S997P**		**X514_splice**; **R533Dfs*3 (Gain)**; **K9Sfs*10**; D1020Y; F514L; **R533Kfs*8**; **R934W**; **R947Q**; D683E; **K927T (Gain)**; S16Y; S933L; S657N; **G712E**; **T704K**; R782H; T550A; I564T; D932E
Non-Small Cell Lung Cancer	**A48S (Amp)**; E196D; **E197K (Gain)**; **W456***; A325T; K327N; **G449***; **R391L**; G193V (Gain); C221W (Gain)	**Q519R**; **R523P (Gain)**	**G624R**; **P639T**	L856V (Shallow del); **P866A (Gain)**	E959Q (Gain); V1019L	**R1074* (Gain)**	S607I; A495V (Gain)
Esophagogastric Cancer	D215N; **A345Cfs*18**; **P93L (Gain)**; **G467R (Gain)**; **F344Lfs*5**			**D732G**			**K677Rfs*58**; **P883Q (Gain)**; **R533Dfs*3**; **R546W;** F935L
Melanoma	**T355P**; **W419* (Shallow del)**; **P87L**; **L339P (Gain)**; **P93H**; **Q352K**; W234L; L490M; **R364S (Shallow del); R120S (Shallow del)**; S164F; **H443Y (Shallow del)**	**R523***		G851V; **E818* (Gain)**; W722L (Gain)	S1033F; **H1021Y**; L990F (Gain); L1064I		**P589L**; S583L; **P498Q;** T616I (Gain); **K507N (Shallow del)**; **R506M; P618S**
Breast Cancer	R212K; **D144H**; **X119_splice**			**X832_splice**	R954K; G1069V (Gain)	**K1078N (Gain)**	**E710del**; **R533I (Gain)**; **E574K**; **K677Rfs*58**; E591Q

Pancreatic Cancer	E360D (Gain); A303V; **G238R**; **I311K**; A399P; **R272C**; **X226_splice**; E360D	**R523***			**R947* (Deep del)**; F976C		T557A (Shallow del); **X514_splice**; **R533Dfs*3**; L881I; **E515K (Deep del)**; A698V; E508D
Head and Neck Cancer	**E196***		E623Q				H586R; E78Q (Gain)
Cervical Cancer	R297K; R461H						**S1070* (Gain)**; **P618T (Shallow del)**
Pleural Mesothelioma							**P694R**

Table S2. All ZRANB3 mutations retrieved from TCGA Pan Cancer database [18] grouped in each cancer type and relative to each functional domain. Mutations in bold are characterized by a putative critical or severe functional substitution of the original residue; some of these associates with Copy Number Alterations/Variations (CNAs/CNVs, red marked) different than diploid.

Cancer Type	n_wt	rho_wt	p_wt	n_mut	rho_mut	p_mut	D rho (mut-wt)
All *	4488	**0,387**	0,000	5236	**0,184**	0,000	**-0,203**
ACC	46	**0,713**	0,000	28	0,227	0,245	**-0,486**
BLCA	111	**0,281**	0,003	280	**0,167**	0,005	-0,114
BRCA *	355	**0,342**	0,000	693	**0,125**	0,001	**-0,217**
BRCA.Basal	20	0,008	0,972	170	0,096	0,212	0,088
BRCA.Her2	9	**0,769**	0,015	73	0,060	0,617	**-0,709**
BRCA.LumA	264	**0,403**	0,000	301	**0,282**	0,000	-0,121
BRCA.LumB *	52	**0,391**	0,004	165	**0,163**	0,037	**-0,228**
BRCA.Normal	23	**0,717**	0,000	17	**0,764**	0,000	0,046
CESC	184	**0,432**	0,000	102	**0,249**	0,012	-0,182
CHOL	19	-0,071	0,772	12	-0,014	0,964	0,057
COAD	148	**0,317**	0,000	309	-0,037	0,516	**-0,354**
DLBC	30	**0,451**	0,012	12	-0,022	0,945	**-0,473**
ESCA	16	0,145	0,592	156	**0,301**	0,000	0,156
GBM	223	0,069	0,303	117	0,114	0,220	0,045
HNSC *	125	**0,568**	0,000	371	**0,297**	0,000	**-0,271**
KICH	14	0,499	0,069	47	0,070	0,642	**-0,430**
KIRC	455	**0,527**	0,000	68	**0,427**	0,000	-0,100
KIRP	103	**0,539**	0,000	172	**0,578**	0,000	0,039
LAML	138	**0,426**	0,000	11	-0,229	0,499	**-0,654**
LGG	253	**0,262**	0,000	230	**0,316**	0,000	0,054
LIHC	133	**0,407**	0,000	203	**0,363**	0,000	-0,044
LUAD	153	**0,302**	0,000	343	0,052	0,341	**-0,250**
LUSC	47	**0,078**	0,602	434	**0,250**	0,000	0,172
MESO	49	**0,538**	0,000	31	0,149	0,425	**-0,389**
OV	59	-0,137	0,302	347	**0,204**	0,000	**0,341**
PAAD	56	0,097	0,477	118	**0,362**	0,000	**0,265**

PCPG	106	**0,379**	0,000	68	0,124	0,316	**-0,255**
PRAD *	314	**0,489**	0,000	157	**0,219**	0,006	**-0,271**
READ	26	**0,427**	0,030	133	0,066	0,452	**-0,361**
SARC	84	**0,322**	0,003	159	-0,079	0,323	**-0,401**
SKCM	59	**0,354**	0,006	34	0,195	0,269	-0,159
STAD	153	**0,346**	0,000	239	**0,197**	0,002	-0,149
TGCT	76	0,163	0,160	66	-0,150	0,229	**-0,313**
THCA	460	**0,533**	0,000	19	0,352	0,139	-0,181
THYM	102	**0,271**	0,006	16	0,121	0,655	-0,149
UCEC *	315	**0,595**	0,000	207	**0,228**	0,001	**-0,366**
UCS	5	-0,853	0,066	46	-0,101	0,503	**0,752**
UVM	71	-0,025	0,837	8	-0,124	0,771	-0,099

Table S3. Co-expression of *ZRANB3* and *TP53* across all TCGA tumours [18]. Data obtained from linear fit are shown, particularly the correlation coefficients rho with each correspondent p-value and their difference (Δrho) between rho_mut (refers to the correlation between *ZRANB3* and *TP53* mutated) and rho_wt (refers to the correlation between *ZRANB3* and *TP53* wt). Those highlighted in bold are statistically significant rho (p-value < 0.05). In bold are also Δrho > 0.2 or < -0.2. Cancers with significant rho_wt and rho_mut together, and with Δrho > 0.2 or < -0.2 are indicated in red and with *.

**Cancer Type**	**TP53**	**TP53**	**TP53.mutyes**	**TP53.mutyes**	**MKI67**	**MKI67**	**TP53:TP53. mutyes**	**TP53:TP53. mutyes**	**TP53:MKI67**	**TP53:MKI67**	**TP53.mutyes: MKI67**	**TP53.mutyes: MKI67**
	**(b)**	**(p)**	**(b)**	**(p)**	**(b)**	**(p)**	**(b)**	**(p)**	**(b)**	**(p)**	**(b)**	**(p)**
**ACC**	**8.15E-02**	5.58E-07	9.66E-01	8.11E-02	5.29E-02	1.10E-01	-2.91E-02	3.74E-01	-2.04E-03	1.53E-01	5.12E-03	7.99E-01
**BLCA**	6.19E-03	1.50E-01	4.06E-02	8.81E-01	**2.86E-02**	3.93E-07	3.94E-03	4.02E-01	**-1.45E-04**	4.84E-02	-6.15E-03	1.70E-01
**BRCA ***	**1.77E-02**	2.58E-07	1.97E-01	3.84E-01	**2.55E-02**	1.21E-07	**-8.48E-03**	2.83E-02	**-1.60E-04**	2.30E-03	-1.68E-03	6.68E-01
**CESC**	**2.08E-02**	4.10E-04	5.41E-01	1.17E-01	**1.90E-02**	5.79E-04	-4.75E-03	3.49E-01	-7.95E-05	3.46E-01	6.35E-05	9.89E-01
**CHOL**	-1.93E-02	1.27E-01	1.11E+00	1.56E-01	-1.32E-02	7.49E-01	-1.22E-02	4.53E-01	**1.63E-03**	1.61E-02	-4.91E-02	8.55E-02
**COAD**	4.50E-03	3.14E-01	**7.92E-01**	2.69E-02	**1.92E-02**	4.20E-04	**-1.08E-02**	7.48E-03	-3.70E-05	4.66E-01	4.23E-04	9.17E-01
**DLBC**	-7.96E-03	8.44E-01	8.30E-01	8.08E-01	4.29E-03	9.22E-01	2.98E-03	9.49E-01	1.44E-04	7.76E-01	-2.44E-02	6.79E-01
**ESCA**	7.44E-03	6.77E-01	-1.98E-01	7.77E-01	3.57E-03	7.83E-01	-1.10E-03	9.51E-01	-3.84E-05	5.15E-01	1.47E-02	2.53E-01
**GBM**	1.77E-02	9.95E-02	4.86E-01	5.03E-01	**2.27E-01**	3.20E-19	-9.15E-03	5.77E-01	**-2.11E-03**	2.15E-07	-2.14E-02	3.65E-01
**HNSC ***	**1.95E-02**	2.17E-08	1.39E-01	3.91E-01	**1.50E-02**	1.20E-06	-3.76E-03	2.54E-01	**-1.45E-04**	4.03E-05	3.57E-03	1.93E-01
**KICH**	**5.83E-02**	1.37E-05	2.75E-01	8.30E-02	7.52E-02	2.89E-01	**-5.36E-02**	1.01E-03	1.87E-03	2.19E-01	-5.67E-02	4.07E-01
**KIRC**	**2.95E-02**	3.25E-09	**-5.31E-01**	3.29E-02	**4.83E-02**	2.25E-03	**2.10E-02**	2.31E-02	-6.72E-04	6.10E-02	-1.18E-02	3.34E-01
**KIRP**	**3.13E-02**	1.97E-09	-6.57E-02	7.12E-01	1.67E-02	4.61E-01	-4.21E-03	4.69E-01	6.11E-04	1.77E-01	-6.29E-04	9.72E-01
**LAML**	**9.70E-02**	1.34E-05	2.38E+00	3.04E-01	**3.99E-02**	3.37E-02	**-1.53E-01**	9.35E-03	-5.54E-04	6.27E-02	3.21E-02	1.86E-01
**LGG**	4.22E-03	1.38E-01	-1.74E-01	1.88E-01	1.07E-02	3.01E-01	-3.52E-03	3.70E-01	**4.56E-04**	9.53E-03	**1.64E-02**	1.63E-02
**LIHC**	**2.53E-02**	1.57E-03	2.19E-01	1.46E-01	**2.30E-02**	1.94E-02	2.32E-03	7.94E-01	-7.72E-05	8.34E-01	-4.02E-03	6.55E-01
**LUAD**	-4.81E-04	9.38E-01	6.03E-01	5.24E-02	1.85E-03	8.36E-01	-1.39E-02	5.44E-02	**3.33E-04**	1.42E-02	1.78E-03	8.16E-01
**LUSC**	3.74E-03	5.11E-01	-7.70E-02	8.20E-01	**2.49E-02**	4.42E-06	8.54E-03	1.35E-01	**-1.36E-04**	3.72E-02	1.34E-03	8.30E-01
**MESO**	**2.80E-02**	1.25E-03	1.17E+00	6.36E-02	**5.26E-02**	1.14E-02	-1.48E-02	1.97E-01	-6.15E-04	5.28E-02	-4.35E-03	7.76E-01
**OV**	4.19E-03	3.35E-01	-6.63E-02	8.85E-01	**2.47E-02**	1.37E-02	1.42E-03	7.41E-01	-5.13E-05	4.37E-01	-5.47E-04	9.56E-01
**PAAD**	6.60E-03	3.53E-01	**-7.46E-01**	1.72E-03	**2.96E-02**	3.60E-02	**1.87E-02**	2.58E-02	**-6.68E-04**	4.57E-02	5.32E-03	6.13E-01
**PCPG ***	**8.55E-02**	1.92E-06	**7.12E-01**	1.27E-02	**4.50E-01**	1.22E-04	**-5.40E-02**	2.97E-02	**-1.86E-02**	2.35E-03	-2.01E-02	8.04E-01
**PRAD ***	**3.97E-02**	1.19E-13	**8.54E-01**	2.47E-03	**1.29E-01**	6.81E-05	-1.04E-02	1.84E-01	**-1.85E-03**	2.29E-03	-3.08E-02	1.17E-01
**READ**	7.49E-03	2.14E-01	-2.13E-01	6.85E-01	7.90E-03	4.78E-01	-6.99E-03	2.49E-01	-1.80E-05	7.54E-01	9.48E-03	3.49E-01
**SARC**	**1.56E-02**	4.75E-02	6.48E-01	1.13E-01	7.38E-03	3.77E-01	-1.11E-02	1.86E-01	1.80E-04	1.49E-01	2.56E-03	7.27E-01
**SKCM**	1.32E-02	2.50E-01	3.07E-01	5.45E-01	2.59E-02	3.25E-01	-2.57E-03	7.86E-01	1.46E-04	7.75E-01	4.57E-03	7.87E-01

**STAD**	4.17E-03	3.18E-01	-3.41E-01	1.07E-01	**1.59E-02**	6.00E-06	5.56E-03	1.96E-01	-7.43E-05	8.53E-02	2.97E-03	3.07E-01
**TGCT**	5.28E-02	1.24E-01	2.13E+00	2.40E-01	**9.84E-02**	3.11E-03	**-6.96E-02**	1.47E-02	-5.39E-04	3.38E-01	2.71E-02	1.71E-01
**THCA**	**5.43E-02**	7.23E-20	5.42E-01	1.90E-01	-1.60E-02	8.35E-01	**-2.72E-02**	4.19E-02	-9.90E-04	6.49E-01	**1.69E-01**	4.11E-02
**THYM**	-1.35E-02	1.20E-01	5.05E-01	5.25E-01	1.20E-03	9.06E-01	5.17E-03	7.46E-01	1.47E-04	1.54E-01	-1.33E-02	4.56E-01
**UCEC ***	**2.19E-02**	6.79E-11	**9.49E-01**	4.11E-03	**7.29E-02**	2.33E-16	**-7.65E-03**	4.40E-02	**-3.75E-04**	1.35E-08	-7.10E-03	2.92E-01
**UCS**	-4.33E-02	2.49E-01	-5.06E+00	6.34E-02	-2.27E-02	8.34E-01	3.22E-02	4.12E-01	1.54E-04	7.53E-01	3.02E-02	7.58E-01
**UVM**	-1.43E-02	2.90E-01	-8.21E-01	3.79E-01	8.15E-02	4.91E-01	-2.38E-03	9.03E-01	4.89E-04	8.21E-01	1.36E-01	3.69E-01

Table S4. Multivariate analysis of *ZRANB3* expression with respect to *TP53* and *MKI-67* expressions across TCGA tumours and as function of cell proliferation (expression of *MKI-67*) [18]. The coefficients (b) and the correspondent p-values (p) of each term are reported. The three terms discussed in the main text are highlighted in yellow (TP53, MKI67, and TP53:MKI67). Positive values are in green, negative in orange. Values in bold have statistical significance (p-value < 0.05). In BRCA, HNSC, PCPG, PRAD, and UCEC, but not in the other tumours, these significant values are present in all the terms above.

References

[1] S.F. Altschul, T.L. Madden, A.A. Schäffer, J. Zhang, Z. Zhang, W. Miller, D.J. Lipman, Gapped BLAST and PSI-BLAST: a new generation of protein database search programs, Nucleic Acids Res 25 (1997) 3389–3402. https://doi.org/10.1093/nar/25.17.3389.

[2] P. Jones, D. Binns, H.-Y. Chang, M. Fraser, W. Li, C. McAnulla, H. McWilliam, J. Maslen, A. Mitchell, G. Nuka, S. Pesseat, A.F. Quinn, A. Sangrador-Vegas, M. Scheremetjew, S.-Y. Yong, R. Lopez, S. Hunter, InterProScan 5: genome-scale protein function classification, Bioinformatics 30 (2014) 1236–1240. https://doi.org/10.1093/bioinformatics/btu031.

[3] S.C. Dyer, O. Austine-Orimoloye, A.G. Azov, M. Barba, I. Barnes, V.P. Barrera-Enriquez, A. Becker, R. Bennett, M. Beracochea, A. Berry, J. Bhai, S.K. Bhurji, S. Boddu, P.R. Branco Lins, L. Brooks, S.B. Ramaraju, L.I. Campbell, M.C. Martinez, M. Charkhchi, L.A. Cortes, C. Davidson, S. Denni, K. Dodiya, S. Donaldson, B. El Houdaigui, T. El Naboulsi, O. Falola, R. Fatima, T. Genez, J.G. Martinez, T. Gurbich, M. Hardy, Z. Hollis, T. Hunt, M. Kay, V. Kaykala, D. Lemos, D. Lodha, N. Mathlouthi, G.A. Merino, R. Merritt, L.P. Mirabueno, A. Mushtaq, S.N. Hossain, J.G. Pérez-Silva, M. Perry, I. Piližota, D. Poppleton, I. Prosovetskaia, S. Raj, A.I.A. Salam, S. Saraf, N. Saraiva-Agostinho, S. Sinha, B. Sipos, V. Sitnik, E. Steed, M.-M. Suner, L. Surapaneni, K. Sutinen, F.F. Tricomi, I. Tsang, D. Urbina-Gómez, A. Veidenberg, T.A. Walsh, N.L. Willhoft, J. Allen, J. Alvarez-Jarreta, M. Chakiachvili, J. Cheema, J.B. da Rocha, N.H. De Silva, S. Giorgetti, L. Haggerty, G.R. Ilsley, J. Keatley, J.E. Loveland, B. Moore, J.M. Mudge, G. Naamati, J. Tate, S.J. Trevanion, A. Winterbottom, B. Flint, A. Frankish, S.E. Hunt, R.D. Finn, M.A. Freeberg, P.W. Harrison, F.J. Martin, A.D. Yates, Ensembl 2025, Nucleic Acids Research 53 (2025) D948–D957. https://doi.org/10.1093/nar/gkae1071.

[4] J. Wang, F. Chitsaz, M.K. Derbyshire, N.R. Gonzales, M. Gwadz, S. Lu, G.H. Marchler, J.S. Song, N. Thanki, R.A. Yamashita, M. Yang, D. Zhang, C. Zheng, C.J. Lanczycki, A. Marchler-Bauer, The conserved domain database in 2023, Nucleic Acids Research 51 (2023) D384–D388. https://doi.org/10.1093/nar/gkac1096.

[5] R.C. Edgar, MUSCLE: multiple sequence alignment with high accuracy and high throughput, Nucleic Acids Res 32 (2004) 1792–1797. https://doi.org/10.1093/nar/gkh340.

[6] L.-T. Nguyen, H.A. Schmidt, A. von Haeseler, B.Q. Minh, IQ-TREE: A Fast and Effective Stochastic Algorithm for Estimating Maximum-Likelihood Phylogenies, Molecular Biology and Evolution 32 (2015) 268–274. https://doi.org/10.1093/molbev/msu300.

[7] S. Kalyaanamoorthy, B.Q. Minh, T.K.F. Wong, A. von Haeseler, L.S. Jermiin, ModelFinder: fast model selection for accurate phylogenetic estimates, Nat Methods 14 (2017) 587–589. https://doi.org/10.1038/nmeth.4285.

[8] A.A. Neath, J.E. Cavanaugh, The Bayesian information criterion: background, derivation, and applications, WIREs Computational Statistics 4 (2012) 199–203. https://doi.org/10.1002/wics.199.

[9] B.Q. Minh, M.A.T. Nguyen, A. von Haeseler, Ultrafast Approximation for Phylogenetic Bootstrap, Molecular Biology and Evolution 30 (2013) 1188–1195. https://doi.org/10.1093/molbev/mst024.

[10] M. Ljubic, C. D'Ercole, Y. Waheed, A. de Marco, J. Borišek, M. De March, Computational study of the HLTF ATPase remodeling domain suggests its activity on dsDNA and implications in damage tolerance, Journal of Structural Biology 216 (2024) 108149. https://doi.org/10.1016/j.jsb.2024.108149.
[11] M. Varadi, S. Anyango, M. Deshpande, S. Nair, C. Natassia, G. Yordanova, D. Yuan, O. Stroe, G. Wood, A. Laydon, A. Žídek, T. Green, K. Tunyasuvunakool, S. Petersen, J. Jumper, E. Clancy, R. Green, A. Vora, M. Lutfi, M. Figurnov, A. Cowie, N. Hobbs, P. Kohli, G. Kleywegt, E. Birney, D. Hassabis, S. Velankar, AlphaFold Protein Structure Database: massively expanding the structural coverage of protein-sequence space with high-accuracy models, Nucleic Acids Res 50 (2022) D439–D444. https://doi.org/10.1093/nar/gkab1061.
[12] L. Holm, A. Laiho, P. Törönen, M. Salgado, DALI shines a light on remote homologs: One hundred discoveries, Protein Science 32 (2023) e4519. https://doi.org/10.1002/pro.4519.
[13] H.M. Berman, J. Westbrook, Z. Feng, G. Gilliland, T.N. Bhat, H. Weissig, I.N. Shindyalov, P.E. Bourne, The Protein Data Bank, Nucleic Acids Res 28 (2000) 235–242. https://doi.org/10.1093/nar/28.1.235.
[14] E. Krissinel, K. Henrick, Inference of macromolecular assemblies from crystalline state, J Mol Biol 372 (2007) 774–797. https://doi.org/10.1016/j.jmb.2007.05.022.
[15] Pymol: an open-source molecular graphics tool – ScienceOpen, (n.d.). https://www.scienceopen.com/document?vid=4362f9a2-0b29-433f-aa65-51db01f4962f (accessed April 28, 2025).
[16] H. Chai, J. Zhang, G. Yang, Z. Ma, An evolution-based DNA-binding residue predictor using a dynamic query-driven learning scheme, Molecular BioSystems 12 (2016) 3643–3650. https://doi.org/10.1039/C6MB00626D.
[17] J. Zhang, S. Basu, L. Kurgan, HybridDBRpred: improved sequence-based prediction of DNA-binding amino acids using annotations from structured complexes and disordered proteins, Nucleic Acids Research 52 (2024) e10. https://doi.org/10.1093/nar/gkad1131.
[18] E. Cerami, J. Gao, U. Dogrusoz, B.E. Gross, S.O. Sumer, B.A. Aksoy, A. Jacobsen, C.J. Byrne, M.L. Heuer, E. Larsson, Y. Antipin, B. Reva, A.P. Goldberg, C. Sander, N. Schultz, The cBio Cancer Genomics Portal: An Open Platform for Exploring Multidimensional Cancer Genomics Data, Cancer Discov 2 (2012) 401–404. https://doi.org/10.1158/2159-8290.CD-12-0095.
[19] M. Mounir, M. Lucchetta, T.C. Silva, C. Olsen, G. Bontempi, X. Chen, H. Noushmehr, A. Colaprico, E. Papaleo, New functionalities in the TCGAbiolinks package for the study and integration of cancer data from GDC and GTEx, PLOS Computational Biology 15 (2019) e1006701. https://doi.org/10.1371/journal.pcbi.1006701.
[20] Z. Tang, B. Kang, C. Li, T. Chen, Z. Zhang. GEPIA2: an enhanced web server for large-scale expression profiling and interactive analysis. Nucleic Acids Res. 47(1) 2019 W556-W560. https://doi: 10.1093/nar/gkz430.
[21] GDC Data Portal Homepage, (n.d.). https://portal.gdc.cancer.gov/ (accessed June 20, 2025).
